# Kidney biopsy practice amongst Australasian nephrologists

**DOI:** 10.1186/s12882-021-02505-9

**Published:** 2021-08-26

**Authors:** J. P Burke, T Pham, S May, S Okano, S. K Ratanjee, Z Thet, J. K.W Wong, S Venuthurupalli, D Ranganathan

**Affiliations:** 1 Kidney Health Service, Royal Brisbane and Women’s Hospital, Brisbane, QLD Australia; 2 Kidney Health Service, Rockhampton Base Hospital, Rockhampton, QLD Australia; 3 Kidney Health Service, Tamworth Hospital, Tamworth, NSW Australia; 4 Statistics Unit, QIMR Berghofer Medical Research Institute, Herston, QLD Australia; 5 School of Medicine, Griffith University, Brisbane, QLD Australia; 6 School of Medicine, University of Queensland, Brisbane, QLD Australia; 7 Renal Unit, Liverpool Hospital, Liverpool, NSW Australia; 8 Kidney Health Service, Ipswich Hospital, Ipswich, QLD Australia

**Keywords:** Australia, Guidelines, Nephrology, Kidney biopsy, Survey

## Abstract

**Background:**

Percutaneous kidney biopsy is the gold standard investigation for the diagnosis of kidney diseases. The associated risks of the procedure depend on the skill and experience of the proceduralist as well as the characteristics of the patient. The Kidney Health Australia – Caring for Australasians with Renal Impairment (KHA-CARI) guidelines on kidney biopsies, published in 2019, are the only published national kidney biopsy guidelines. As such, this study surveys current kidney biopsy practices in Australasia and examines how they align with the Australian guidelines, as well as international biopsy practice.

**Methods:**

A cross-sectional, multiple-choice questionnaire was developed examining precautions prior to kidney biopsy; rationalisation of medications prior to kidney biopsy; technical aspects of kidney biopsy; complications of kidney biopsy; and indications for kidney biopsy. This was distributed to all members of the Australian and New Zealand Society of Nephrology (ANZSN).

**Results:**

The response rate for this survey is approximately 21.4 % (182/850). Respondents found agreement (> 75.0 %) in only six out of the twelve questions (50.0 %) which assessed their practice against the KHA-CARI guidelines.

**Conclusions:**

This is the first study of its kind where kidney biopsy practices are examined against a clinical guideline. Furthermore, responses showed that practices were incongruent with guidelines and that there was a lack of consensus on many issues.

**Supplementary Information:**

The online version contains supplementary material available at 10.1186/s12882-021-02505-9.

## Background

Percutaneous kidney biopsy is the gold standard investigation for the diagnosis of kidney parenchymal diseases, providing more information to guide decisions for management of kidney disease and discussion with patients regarding their prognosis [1]. Furthermore, histopathological analysis aids clinicians in understanding the mechanism of disease [2]. In the context of kidney transplantation, a kidney biopsy is the gold standard for the diagnosis of acute kidney allograft rejection [3] and can aid in changing the immunosuppressive regimen for patients.

Despite the clinical benefits, the procedure is not without risks. The major risks associated with kidney biopsies pertain to bleeding and its sequelae. Major bleeding is associated with significant pain, haemodynamic instability and urinary obstruction, and may require blood transfusions, or intervention, or even lead to death [1]. The literature however, points to the fact that the kidney biopsy is a relatively safe procedure with 1.6 % of cases requiring a blood transfusion post-biopsy, and 0.3 % requiring angiographic or surgical intervention (nephrectomy) [4]. The estimated frequency of death as a complication of the procedure is < 0.1 % [4, 5].

Recently in late 2019, the Kidney Health Australia – Caring for Australasians with Renal Impairment (KHA-CARI) guidelines on kidney biopsies was published marking the first instance that Australasian guidelines had been published on this topic [6]. The strength of the recommendations in the KHA-CARI guideline were scored (1 or 2) depending on strength of consensus with an assessment of the quality of evidence (A, B C or D) in accordance with the GRADE working group process (www.gradeworkinggroup.org). Some recommendations are ungraded. Considering this, it is important to determine the current standards by which Australasian nephrologists carry out kidney biopsies and to gauge whether these match evidence-based guidelines. Only a few studies internationally have examined kidney biopsy practices overall [7–9], and none have correlated these practices with evidence-based guidelines.

## Methods

### Aim, study design and setting

The aim of the survey is to define current practices amongst nephrologists when deciding to embark upon a kidney biopsy for a patient and attempts to correlate these practices with the current KHA-CARI guidelines. This survey therefore, alongside the Queensland Renal Biopsy Registry (QRBR) data collection [10] (which provides information about age, gender, clinical indication for biopsy and histopathological findings), gives a more complete picture of this area of renal medicine.

This cross-sectional, multiple-choice questionnaire was developed to examine the most common clinical scenarios where a clinician may decide to order a kidney biopsy. Questions were also designed to understand routine technical aspects of the procedure itself, with attention to recent KHA-CARI recommendations for kidney biopsy practice [6]. A first draft of the questions was devised by investigators JB and DR and initially circulated to a group of 5 other nephrologists and trainees for comments and review. The survey was subsequently circulated to 22 nephrologists and advanced trainees as a pilot survey. Where possible, responses were divided into discrete multiple-choice answers to allow quantitative analysis, with options for additional free text entry allowing additional qualitative analysis. After further feedback and modification following the pilot survey, survey questions were then submitted to the Australian and New Zealand Society of Nephrology (ANZSN) Executive Committee for approval. ANZSN referred the questionnaire to the ANZSN interventional nephrology interest group, who provided additional feedback on question structure and wording. A final version of the survey was distributed via the ANZSN email newsletter; answers were submitted anonymously via an on-line survey link. This meant that the survey was only made available to practicing and retired nephrologists and nephrology advanced trainees practicing in Australia and New Zealand. The survey was available for submissions for 10 weeks from 18th December 2019 to 26th February 2020. A reminder email was sent monthly via the ANZSN newsletter to encourage submissions.

### Survey instrument

There were 26 questions in total (see Additional file 1). The questions were estimated to take 10 to 12 min to answer. Survey questions were divided into 5 sections: precautions and laboratory investigations prior to kidney biopsy, rationalisation of medications prior to kidney biopsy, technical aspects of kidney biopsy, complications of kidney biopsy, and indications for kidney biopsy.

Questions regarding precautions prior to kidney biopsy (questions 1–4) included: blood pressure targets, pre-biopsy coagulation profile investigations, haemoglobin targets, and platelet targets. Questions regarding medications (questions 5–8) included: use of desmopressin, antiplatelet agents and DOAC (Direct Oral Anticoagulant) cessation and use of heparin bridging. Questions regarding technical aspects (questions 9–16) included: proceduralist type and skill-level, needle passes, needle gauge, post-biopsy imaging, patient positioning (for both native and transplant kidneys), post-biopsy observation and hospital discharge. An additional question was added assessing the number of glomeruli in an adequate kidney biopsy specimen. Questions regarding kidney biopsy complications (question 17) surveyed estimated rates of specific complications. Questions regarding indications for kidney biopsy (questions 18–26) employed circumstances involving: acute kidney injury (AKI), chronic kidney disease (CKD), glomerulopathy, diabetes mellitus, solitary kidneys, pregnancy, and kidney transplantation.

Questions 4, 6, 11, 12, 14 and 15 were designed to check alignment of current clinical practice with the KHA-CARI guidelines.

### Statistical analysis

Data analysis was conducted by SO (who was not involved in the design or distribution of the survey). Questionnaire data were summarised using descriptive statistics reporting number and percentage. Questions where respondents were asked to indicate the likelihood of kidney biopsy were dichotomised with positive responses encompassing ‘always’ and ‘usually’ responses and negative responses encompassing ‘sometimes’ and ‘rarely’ responses. In order to gauge respondents’ alignment with current guidelines, answers to the questions outlined above were compared with current KHA-CARI kidney biopsy guidelines in order to quantify respondents’ agreement with these recommendations. The rates of agreement were described with 95 % confidence intervals, calculated using logit transformation-based method.

## Results

In total, 182 respondents completed the survey. The ANZSN newsletter was sent to approximately 850 members (who are nephrologists or nephrology advanced trainees). The response rate for this survey is approximately 21.4 % (182/850). While the window for submissions was 10-week, we noted that only 3 submissions were received in the final 4 weeks; this likely indicates that a longer survey window would be unlikely to produce additional responses. Respondents most selected that they performed between 1 and 5 biopsies during an average month (*n* = 71, 39.4 %); with 34.4 % (*n* = 62) selecting that they performed no biopsies in an average month.

### Precautions prior to kidney biopsy

Table 1 outlines responses relating to pre-biopsy precautions. 56.6 % (*n* = 103) of respondents found a blood pressure of > 160/90 unsafe for biopsy. For baseline blood tests ordered before biopsy, 97.8 % (*n* = 178) would order an INR, 93.4 % (*n* = 170) a full blood count, and 91.2 % (*n* = 166) an APTT. Additionally, 20 respondents (11.0 %) added that they would order kidney function. The most selected haemoglobin cut-off prior to biopsy was 90 g/L (*n* = 44, 24.2 %) with “no target” next most selected (*n* = 36, 19.8 %). The most selected platelet cut-off was 100 × 10^9^/L (*n* = 95, 52.2 %).

**Table 1:** Precautions prior to renal biopsy (*N* = 182)

	n (%)
Blood pressure limit as contraindication to biopsy	
No limit	12 (6.6)
> 180/90mmHg	12 (6.6)
> 160/90mmHg	103 (56.6)
At most > 150/90mmHg	45 (24.7)
Other	10 (5.5)
Blood tests ordered prior to biopsy	
Full blood count	170 (93.4)
International normalised ratio (INR)	178 (97.8)
Activated partial thromboplastin time (APTT)	166 (91.2)
Kidney function tests (urea, electrolytes, creatinine)	20 (11.0)
Bleeding time	15 (8.2)
Minimum Haemoglobin target prior to biopsy	
> 110 g/L	3 (1.6)
> 100 g/L	30 (16.5)
> 90 g/L	44 (24.2)
> 80 g/L	30 (16.5)
> 70 g/L	21 (11.5)
No target	36 (19.8)
Target depends on the clinical scenario	15 (8.2)
Other	3 (1.6)
Minimum Platelet target prior to biopsy	
> 100 × 10^9^/L	95 (52.2)
> 50 × 10^9^/L	71 (39.0)
> 20 × 10^9^/L	1 (0.5)
Other	15 (8.2)

### Medications prior to biopsy

Respondents selected an eGFR of < 15 most commonly (*n* = 57, 31.5 %) as indication for desmopressin (DDAVP) but close to a quarter of respondents did not use desmopressin at all (*n* = 42, 23.2 %). Those who did not use eGFR as indication for DDAVP used uraemia instead with cut-offs ranging from 20 to 40mmol/L (Table 2). Most would withhold aspirin for at least 7 days prior to biopsy (*n* = 108, 59.3 %) in cases where the patient had low risk of a cardiovascular event and would not withhold aspirin (*n* = 103, 56.6 %) if the patient had a higher risk of a cardiovascular event. Most respondents withheld P2Y12 inhibitors (e.g. Clopidogrel) for 7 days prior to biopsy (*n* = 106, 58.2 %) and DOACs for 3 days prior to biopsy (*n* = 73, 40.1 %). For bridging heparin, most opted to cease infusions 6 h before (*n* = 98, 53.8 %) and to restart infusions at least 24 h after (*n* = 78, 43.1 %) a biopsy.

**Table 2:** Medications prior to renal biopsy (*N* = 182)

	n (%)
Conditions for giving DDAVP prior to renal biopsy (*n* = 181)	
Do not give DDAVP	42 (23.2)
If eGFR < 30	31 (17.1)
If eGFR < 15	57 (31.5)
Uraemia (with varying urea cut-offs)	33 (18.2)
Other	18 (9.9)
Days before renal biopsy that the following medications would be withheld:	
Aspirin (low risk of cardiovascular event)	
Would not withhold	19 (10.4)
3 days	5 (2.7)
5 days	50 (27.5)
7 days	96 (52.7)
10 + days	12 (6.6)
Aspirin (high risk of cardiovascular event)	
Would not withhold	103 (56.6)
3 days	20 (11.0)
5 days	26 (14.3)
7 days	32 (17.6)
10 + days	1 (0.6)
P2Y12 inhibitors (clopidogrel, ticagrelor, etc.)	
Would not withhold	5 (2.7)
3 days	4 (2.2)
5 days	50 (27.5)
7 days	106 (58.2)
10 + days	17 (9.3)
Direct oral anticoagulants (apixaban, rivaroxaban, etc.)	
Would not withhold	3 (1.6)
3 days	73 (40.1)
5 days	57 (31.3)
7 days	42 (23.1)
10 + days	7 (3.8)
Time when bridging intravenous heparin is ceased before biopsy	
4 h beforehand	40 (22.0)
6 h beforehand	98 (53.8)
At least 10 h beforehand	37 (20.3)
Other	7 (3.8)
Time when bridging intravenous heparin is restarted after biopsy (*n* = 181)	
6 h after biopsy	47 (26.0)
12 h after biopsy	35 (19.3)
At least 24 h after biopsy	78 (43.1)
Depends on clinical context (risk of thrombosis/bleeding, etc.)	16 (8.8)
Other	5 (2.8)

### Technical aspects of kidney biopsy

Most respondents preferred senior training physicians to perform the kidney biopsy (*n* = 108, 59.3 %) (Table 3). The most selected maximum number of passes taken when performing a biopsy was split between 3 passes (*n* = 70, 38.5 %) and 4 passes (*n* = 69, 37.9 %). The 16-gauge needle was most selected in both allograft (*n* = 104, 58.1 %) and native (*n* = 122, 67.8 %) kidney biopsies. Prone positioning was most selected for native kidney biopsies (*n* = 156, 86.7 %) and supine positioning for transplant kidney biopsies (*n* = 161, 89.9 %). Approximately 90 % of respondents (*n* = 163) treated kidney biopsies as day cases, with most (89.6 %) opting to observe patients for 4 to 6 h post-procedure.

**Table 3:** Technical aspects of renal biopsy procedure (*N* = 182)

	n (%)
Average number of biopsies performed in one month (*n* = 180)	
None	62 (34.4)
1–5	71 (39.4)
6–10	30 (16.7)
> 10	17 (9.4)
Prefer renal biopsies to be conducted by	
Ultra-sonographers	3 (1.6)
Senior Renal Registrars	108 (59.3)
Consultant nephrologists	38 (20.9)
Radiologists	33 (18.1)
Maximum number of passes made during a biopsy	
2	11 (6.0)
3	70 (38.5)
4	69 (37.9)
At least 5	23 (12.6)
Other	9 (4.9)
Needle size for biopsy if:	
Allograft kidney biopsy (*n* = 179)^a^	
14 gauge	25 (14.0)
16 gauge	104 (58.1)
18 gauge	52 (29.1)
Native kidney biopsy (*n* = 180)^b^	
14 gauge	35 (19.4)
16 gauge	122 (67.8)
18 gauge	26 (14.4)
Routine imaging post renal biopsy?	
No	173 (95.1)
Yes	9 (4.9)
Adequate number of glomeruli in a biopsy specimen	
8	40 (22.0)
12	58 (31.9)
15	34 (18.7)
20	22 (12.1)
Depends on clinical context	16 (8.8)
Other	12 (6.6)
Position of patient for:	
Native kidney (*n* = 180)	
Supine position	18 (10.0)
Prone position	156 (86.7)
Lateral decubitus/lateral recumbent position	6 (3.3)
Transplant kidney (*n* = 179)	
Supine position	161 (89.9)
Prone position	13 (7.3)
Sitting position	1 (0.6)
Lateral decubitus/lateral recumbent position	4 (2.2)
Length of patient stay in hospital for observation	
4 h	73 (40.1)
6 h	90 (49.5)
8 h	2 (1.1)
At least 12 h	11 (6.0)
Other	6 (3.3)

^a^2 respondents chose multiple answers

^b^3 respondents chose multiple answers

### Complications of kidney biopsy

Rates for each of the listed possible complications of kidney biopsy (drop in haemoglobin, bleeding requiring transfusion/embolization, nephrectomy, urinary tract infection and death) were overall estimated to be low by responders (Table 4). For bleeding requiring transfusion (*n* = 126, 69.2 %), bleeding requiring embolization (*n* = 157, 86.3 %), urinary tract infection (*n* = 165, 90.7 %), nephrectomy (*n* = 179, 98.4 %) and death (*n* = 180, 98.9 %), the most commonly estimated complication rate was < 1 %.

**Table 4:** Complications of renal biopsy (*N* = 182)

	n (%)
Decrease in haemoglobin > 10 g/L	
< 1 %	81 (44.5)
1–5 %	78 (42.9)
5–10 %	13 (7.1)
> 10 %	10 (5.5)
Bleeding requiring transfusion	
< 1 %	126 (69.2)
1–5 %	52 (28.6)
5–10 %	2 (1.1)
> 10 %	2 (1.1)
Bleeding requiring embolisation	
< 1 %	157 (86.3)
1–5 %	23 (12.6)
5–10 %	0 (0)
10–25 %	2 (1.1)
Urinary tract infection	
< 1 %	165 (90.7)
1–5 %	14 (7.7)
5–10 %	1 (0.5)
> 10 %	2 (1.1)
Nephrectomy	
< 1 %	179 (98.4)
1–5 %	1 (0.5)
5–10 %	0 (0)
10–25 %	2 (1.1)
Death	
< 1 %	180 (98.9)
1–5 %	0 (0)
5–10 %	0 (0)
10–25 %	2 (1.1)

### Indications for kidney biopsy

Answers were varied for indications of kidney biopsy (Table 5). Overall, respondents were in favour of ordering a kidney biopsy when investigations are suggestive of acute glomerulonephritis (*n* = 165, 90.7 %). Respondents were also likely to biopsy if patients had an AKI with elevated ANCA titres (*n* = 173, 95.6 %) or elevated anti-DNAse B titres (*n* = 155, 85.6 %). When considering a patient with a non-recovering AKI, the most selected duration to wait before biopsy was 2 weeks (*n* = 76, 42.9 %). When evaluating a patient with chronic renal insufficiency and reduced kidney size on imaging, only 2.2 % (*n* = 4) of respondents chose to biopsy. Haematuria and proteinuria > 1 g/day in chronic renal insufficiency was a more favourable indication (*n* = 152, 83.5 %) than haematuria and < 1 g/day proteinuria in chronic renal insufficiency (*n* = 70, 38.5 %).

**Table 5:** Indications for renal biopsy (*N* = 182)

	n (%)
Would order a renal biopsy in the following situations:	
AKI	
When other investigations are suggestive of acute GN	165 (90.7)
Presenting as acute GN with elevated ANCA titres (*n* = 181)	173 (95.6)
Presenting as acute GN with elevated anti-DNAse titres (*n* = 181)	155 (85.6)
CKD (GFR < 30) with unknown cause	
Normal kidney size on imaging	86 (47.3)
Reduced kidney size on imaging	4 (2.2)
Haematuria and proteinuria (> 1 g/day)	152 (83.5)
Haematuria and < 1 g/day of proteinuria	70 (38.5)
Normal renal function	
Haematuria and proteinuria (> 1 g/day) (*n* = 181)	151 (83.4)
Proteinuria (> 1 g/day), without haematuria and normotensive (*n* = 181)	109 (60.2)
Proteinuria (> 1 g/day), without haematuria and hypertensive (*n* = 180)	100 (55.6)
Isolated proteinuria > 3 g/day (*n* = 180)	167 (92.8)
Isolated haematuria and normotensive (*n* = 180)	13 (7.2)
Type 2 diabetes mellitus	
Chronic renal insufficiency (eGFR < 30 mL/min) without retinopathy	20 (11.0)
Rapidly deteriorating renal function	86 (47.3)
Nephrotic range proteinuria	39 (21.4)
Active urinary sediment	126 (69.2)
Solitary kidney	
Abnormal renal function (GFR < 30mL/min)	3 (1.6)
Proteinuria 1-3 g/day	15 (8.2)
Proteinuria > 3 g/day	45 (24.7)
Pregnancy < 32 weeks	
GFR < 30mL/min of unknown cause	31 (17.0)
Symptomatic proteinuria > 3 g/day without clinical features of pre-eclampsia	41 (22.5)
Symptomatic proteinuria > 3 g/day with clinical features of pre-eclampsia	7 (3.8)
Pregnancy ≥ 32 weeks	
GFR < 30mL/min of unknown cause	6 (3.3)
Symptomatic proteinuria > 3 g/day without clinical features of pre-eclampsia	7 (3.8)
Symptomatic proteinuria > 3 g/day with clinical features of pre-eclampsia	3 (1.6)
Transplanted kidney	
Rapid rise in serum creatinine after initially good function, before anti-rejection therapy (*n* = 181)	173 (95.6)
Serum creatinine not improving after anti-rejection therapy	160 (87.9)
Slow progressive deterioration in graft function	90 (49.5)
New onset proteinuria > 3 g/day	161 (88.5)
Number of weeks of non-recovery after AKI when respondent would biopsy (*n* = 177)	
2 weeks	76 (42.9)
4 weeks	57 (32.2)
6 weeks	29 (16.4)
8 weeks	15 (8.5)

*AKI* Acute kidney injury, *CKD* Chronic kidney disease, *GN* Glomerulonephritis

In patients with normal kidney function, isolated proteinuria > 3 g/day (*n* = 167, 92.8 %), as well as proteinuria > 1 g/day and haematuria (*n* = 151, 83.4 %) were favourable indications for biopsy. Respondents were less likely to request a kidney biopsy in patients with isolated proteinuria > 1 g/day independent of their blood pressure status – normotensive (*n* = 109, 60.2 %), hypertensive (*n* = 100, 55.6 %).

In diabetic patients, chronic kidney insufficiency without retinopathy (*n* = 20, 11.0 %) and nephrotic range proteinuria (*n* = 39, 21.4 %) were not seen as a usual indication for kidney biopsy. Conversely, rapidly deteriorating kidney function (*n* = 86, 47.3 %) or the presence of active urinary sediment (*n* = 126, 69.2 %) were more common kidney biopsy indications.

Overall, respondents were less likely to order a kidney biopsy if the patient had a solitary kidney with 45 respondents (24.7 %) opting for a kidney biopsy in patients with proteinuria > 3 g/day.

Respondents were less likely to biopsy pregnant patients. Nearly a quarter (22.5 %, *n* = 41) of respondents would biopsy pregnant patients at less than 32 weeks gestation with proteinuria > 3 g without clinical features of pre-eclampsia. In pregnant patients at later stages of pregnancy (at or greater than 32 weeks gestation), the proportion of respondents that would biopsy decreased (*n* = 7, 3.8 %).

In patients with a kidney transplant, most respondents would biopsy patients with rapidly rising creatinine after good initial kidney function (*n* = 173, 95.6 %). Similarly, kidney biopsy was usually considered in a transplant patient with serum creatinine not improving after anti-rejection therapy (*n* = 160, 87.9 %) and with new onset proteinuria > 3 g/day (*n* = 161, 88.5 %). Fewer respondents considered kidney biopsy in a transplant patient with slowly progressive deteriorating graft function (*n* = 90, 49.5 %).

### Alignment with KHA-CARI guidelines

Table 6 compared the responses to questions which related to the recently released KHA-CARI Renal Biopsy Guidelines. Respondents’ practices differed with the guidelines in their platelet target before biopsy. 39.0 % agreed with the target threshold of > 50 × 10^9^/L (*n* = 71, 95 % confidence interval (CI): 31.9–46.2 %). Half of the respondents however, selected a *higher* platelet target, with 52.2 % (*n* = 95) opting for a target of > 100 × 10^9^/L. 56.6 % of respondents (*n* = 103, 95 % CI: 49.3–63.9 %) followed guideline recommendations to not withhold aspirin in patients with high risk of a cardiovascular event and 40.1 % (*n* = 73, 95 % CI: 32.9–47.3 %) withheld DOACs for 2–3 days prior to biopsy. Regarding needle gauge size; 58.1 % of respondents opted for 16 g in native kidneys (*n* = 104, 95 % CI: 50.8–65.4 %) and 67.8 % (*n* = 122, 95 % CI: 60.9–74.7 %) in allograft kidneys. Finally, 50.5 % (*n* = 92, 95 % CI: 43.2–57.9 %) agreed with the guidelines to observe routine biopsies for 6 to 8 h.

**Table 6:** Agreement of respondents with KHA-CARI Guideline recommendations intervals (*N* = 182)

	Grade of Recommendation^a^	Recommendation	n	%	95 % CI
Platelet target before biopsy	Ungraded	> 50 × 10^9^/L	71	39.0	31.9–46.2
Withholding of anticoagulation and antiplatelet agents					
Aspirin in patient with low risk of cardiovascular event	1 C	3–7 days	151	83.0	77.5–88.5
Aspirin in patient with high risk of cardiovascular event	1 C	No cessation	103	56.6	49.3–63.9
P2Y12 inhibitors (clopidogrel, ticagrelor, etc.)	Ungraded	5–7 days	156	85.7	80.6–90.8
Direct oral anticoagulants (apixaban, rivaroxaban, etc.)	Ungraded	2–3 days	73	40.1	32.9–47.3
Bridging intravenous heparin	Ungraded	4–6 h	138	75.8	69.5–82.1
Renal biopsy needle choice					
Allograft kidney biopsy (*n* = 179)	2 C	16 gauge	104	58.1	50.8–65.4
Native kidney biopsy (*n* = 180)	2 C	16 gauge	122	67.8	60.9–74.7
Post-biopsy imaging	2 C	No routine imaging	173	95.1	91.9–98.2
Positioning of patient					
Native kidney (*n* = 180)	2B	Prone position	156	86.7	81.7–91.7
Transplant kidney (*n* = 179)	Ungraded	Supine position	161	89.9	85.5–94.4
Length of observation post-biopsy	1B	6–8 h	92	50.5	43.2–57.9

^a^Grade of each recommendation and level of evidence, as reported in the KHA-CARI Guideline

There was found to be high levels of agreement (> 75 %) with the guidelines in the following categories: withholding aspirin in patients with low risk of cardiovascular event for 3–7 days (*n* = 151, 83.0 %, 95 % CI: 77.5–88.5 %), withholding P2Y12 inhibitors for 5–7 days (*n* = 156, 85.7 %, 95 % CI: 80.6–90.8 %), withholding bridging intravenous heparin for 4–6 h (*n* = 138, 75.8 %, 95 % CI: 69.5–82.1 %), no routine post-biopsy imaging (*n* = 173, 95.1 %, 95 % CI: 91.9–98.2 %), and the positioning of patients for a native (*n* = 156, 86.7 %, 95 % CI: 81.7–91.7 %) and transplant (*n* = 161, 89.9 %, 95 % CI: 85.5–94.4 %) kidney biopsy.

## Discussion

Our study adds to the currently limited literature examining overall kidney biopsy practices [7–9, 11]. To our knowledge this is the first study that has examined nephrologists’ kidney biopsy practices in comparison to evidence-based guidelines. Our study demonstrates that there is significant variation among Australasian nephrologists surrounding the practice of kidney biopsy. Respondents reported agreement (> 75.0 %) in six out of the twelve questions (50.0 %) which assessed their practice against the KHA-CARI guidelines. Furthermore, the findings of our survey suggest that there are variations in kidney biopsy practices internationally [7, 9].

Given that there was only a short period between this survey (18th December 2019 to 26th February 2020) and the publication of the KHA-CARI guidelines (August 2019), it is plausible that many of the respondents may not have been aware of the guidelines or that the guidelines had not been discussed at a formal level at their health service. The recommendations for practice in the KHA-CARI guidelines were not supported by strong evidence which varied between level B and ‘Ungraded’ (see Table 6). The grading of evidence for recommendations is likely to affect physician concordance. For example, it was noted that the recommendations with lowest rate of physician concordance (platelet target and timing of withholding direct oral anticoagulants) were both ‘Ungraded’ recommendations.

Most respondents (n = 84, 41.0 %) only performed at most five biopsies in an average month and approximately one third did not perform any biopsies. Therefore, the results may reflect the low frequency with which respondents encounter and conduct kidney biopsies. Our dataset did not allow us to conduct subgroup analyses, however, comparing responses between those who more frequently carry out kidney biopsies with those who do so at lesser frequencies would have elucidated whether experience was a confounder in compliance with guidelines. A previous Australian kidney biopsy survey in 2013 by Ritchie et al. [8] found that renal trainees would perform kidney biopsies “most” of the time; and that consultant nephrologists performed kidney biopsies “some” of the time. Given that our survey respondents are likely to be predominantly consultants who carried out lower numbers of kidney biopsies, they may not be representative of the majority of biopsy proceduralists (trainees and interventional radiologists). Nevertheless, consultant nephrologists who order kidney biopsies and manage their complications should have an understanding of the technical aspects of the procedure.

It is our understanding that there has not been a kidney biopsy guideline published in the last 10 years [1]. Therefore, we sought to compare the KHA-CARI guidelines with a recent best-practice review published in the CJASN [1]. When comparing to Hogan et al. and its suggestions, with the KHA-CARI guidelines, the contents are relatively similar. Both suggest that ultrasound guided kidney biopsy is the preferred method of biopsy. Both recommend continuing aspirin for those at high risk for a cardiovascular event, ceasing warfarin 5 days before the procedure and ceasing unfractionated heparin 4–6 h prior to the biopsy. The KHA-CARI guidelines recommend ceasing aspirin 3–7 days prior to the biopsy in patients at a low risk of cardiovascular events whereas American practice is usually 7–10 days. Both recommend no imaging post-biopsy; however, review of American practice suggests a longer period of observation for uncomplicated biopsies (8–12 h vs. 6–8 h).

In terms of the other responses in the survey, there was found to be consensus on the following issues: blood tests ordered prior to a kidney biopsy, the fact that there was no requirement to routinely image the patient after a biopsy, the positioning of a patient for a biopsy and ordering a kidney biopsy for an acute kidney injury (AKI) when patients’ presentations suggest acute glomerulonephritis, when patients present with isolated proteinuria despite normal kidney function and when transplant patients showed signs of graft rejection. Furthermore, there was largely agreement about not carrying out biopsies on pregnant women > 32 weeks with proteinuria and on women with clinical features of pre-eclampsia.

In terms of the complications reported, the responses seemed to match the rates reported in the literature. It has previously been reported in a meta-analysis that 0.9 % of cases require a blood transfusion post-biopsy, 0.6 % requiring angiographic intervention and 0.01 % requiring surgical intervention (nephrectomy) [5]. Most responses in our survey for these complications indicated that the rate was < 1 %. Other studies since the publication of the meta-analysis have reported 1.9 % major complication rates and 5.8 % minor complication rates in an outpatient setting [12] and rates of bleeding ranging from 0.3 to 7.4 % [2, 13]. It is clear therefore, that the rate of complications is dependent upon site-specific variables including: the experience and skill of the person carrying out the procedure, the pre-biopsy biochemical parameters and the population of patients undergoing biopsy. The findings from our survey certainly illustrate this variability as there were still a significant portion of respondents that had indicated > 1 % rate of complications for urinary tract infection and bleeding requiring embolization. Given the clear evidence that the rates for the aforementioned complications are low, this may also suggest respondents’ lack of familiarity with the kidney biopsy procedure. This is echoed in a recent survey of graduate nephrologists in the USA which showed that almost two-thirds of graduates (from 1985 to 2017) no longer performed biopsies and that only half of training program directors believed that nephrologists needed to be competent in kidney biopsies [11].

A previous Australian survey, published in 2013, examined the practices surrounding kidney, surveying 118 nephrology consultants and advanced trainees who were members of the ANZSN [8]. This was a more limited assessment of kidney biopsy practice, examining the remoteness of hospitals in which biopsies were performed, routine pre-biopsy pathology testing and the technical aspects of kidney biopsies. Quite similarly, respondents indicated that they ordered a full blood count, INR and APTT prior to biopsy, that renal trainees mostly performed kidney biopsies, and that most (67.8 %) used a 16-gauge needle to biopsy native kidneys.

In the past decade, there have been two international surveys that have examined nephrologists’ kidney biopsy practices to a similar extent (i.e. pre-biopsy precautions, technical aspects of biopsy, biopsy indications, and complications). The most recent was a 2020 survey of 220 directors of nephrology in Japanese teaching hospitals [7]. When compared with our survey, similar responses were reported for respondents’ indications for biopsy. Respondents agreed that the following were strong indicators for biopsy in adults: haematuria and concomitant proteinuria compared to either feature alone, rapidly progressive glomerulonephritis or AKI, Type 2 diabetes mellitus with concomitant urinary abnormalities. Respondents also were unlikely to biopsy in pregnancy. Furthermore, respondents reported that they too used the 16-gauge needle most commonly and reported similar complication rates post-biopsy. Contrarily, respondents in Kawaguchi et al.’s [7] survey were likely to biopsy patients with isolated haematuria, used a higher blood pressure cut-off (median 180mmHg, IQR: 160-180mmHg), lower platelet cut-off (median: 50 × 10^9^/L) and tended to observe their patients for longer post-biopsy (average length of stay: 4–6 days). The study also assessed the method of processing biopsy specimens which was beyond the scope of our study.

Bollee et al. [9] one decade earlier (2010) surveyed 88 nephrologists and more briefly examined kidney biopsy practices. Similar findings were reported for respondents’ blood tests ordered pre-biopsy (APTT, platelet count), and a similar proportion (58.3 %) of respondents also preferred a 16-gauge needle for biopsy. Respondents however, tended to monitor patients for longer post-biopsy (mean: 24.8 h, SD: 6.9 h) and interestingly many centres (23/27) had residents who performed biopsies; though this may be confounded by differences in practitioner titles. The survey did not examine respondents’ indications for biopsy. Therefore, our study whilst highlighting heterogeneity in the practices of Australasian nephrologists also, in the context of the limited literature available, highlights differences in the practice of nephrologists internationally.

### Strengths and limitations

A strength of this study is the large and encompassing range of questions surrounding the practice of kidney biopsies in Australasia. The questions were also thoroughly examined as the survey questionnaire was developed through an extensive consultative process with a group of expert nephrologists, a pilot survey facilitated by the State-wide Renal Clinical Network, Queensland and a subsequent survey which was endorsed by the ANZSN and its interventional group ANZSIN. It therefore allowed a thorough evaluation of kidney biopsy practices and given the comparison with the KHA-CARI guidelines, is the first survey of its kind. Furthermore, given the limited literature describing kidney biopsy practices, this study adds empirical data with regards to current kidney biopsy practices.

The survey response rate of 21 % is inexact due to difficulty in estimating the number of members who would have even opened the email link; however this rate falls within the range of that expected of an online survey [14]. The survey was distributed solely to members of the ANZSN and therefore excluded nephrologists who are not members. In 2017, at time of the last ANZSN Workforce Report [15] there were 598 nephrologists in Australia and 61 in New Zealand, of which 15.5 % were not members of the ANZSN. It is possible that members and non-members of ANZSN may differ in terms of demographic, academic interests and/or clinical experience; however, this has not been previously studied.

Recall bias, as with all surveys, especially for figures and numbers (for example, complication rates) that are not readily accessible to the respondent could cause conflation of figures. Missing responses were minimal in our study (at most, five responses), therefore minimising potential bias associated with missing data. Furthermore, the lack of demographic data did not allow for further analyses, however, our survey was intended to provide an overview of the current kidney biopsy practices in Australasia as a whole.

It is also acknowledged that the clinical scenarios presented in the survey questions are hypothetical. Responses may vary depending on how the scenario is interpreted by the respondent (based on past experiences); rather than delivering a generalised answer. This is a limitation of presenting a scenario within a survey.

## Conclusions

Our study provides an overview of kidney biopsy practices in Australasia. This is the first study of its kind where kidney biopsy practices are examined against a clinical guideline. Furthermore, responses showed that there was a low level of congruence between clinical practice and the KHA-CARI guidelines and that there was a lack of consensus on many issues relating to kidney biopsies. Our study illustrates the variation within Australasian nephrologists and differences compared with international biopsy practice; and shows that current practice may not always be evidence-based.

## Supplementary Information

**Additional file 1.**

## Data Availability

The datasets generated during and analysed during the current study are not publicly available as survey respondents are potentially identifiable from free text comments in response to questions. However, data are available from the corresponding author on reasonable request.
