# Supplementary material for: Kidney biopsy practice amongst Australasian nephrologists
Source: BMC Nephrol. 2021 Aug 26;22:291. doi: 10.1186/s12882-021-02505-9 (PMC8390249; doi:10.1186/s12882-021-02505-9)
Supplement: Supplementary file 1 — Additional file 1. [file 12882_2021_2505_MOESM1_ESM.docx]

**Renal Biopsy Practice Amongst Australasian Nephrologists**

**Supplementary Material 1: Survey Questions**

| Number | Question | Response |
| --- | --- | --- |
| 1 | What blood pressure limit would be a contraindication to proceeding with renal biopsy? | No limit  >160/90  >150/90  >140/90  >130/90  Other |
| 2 | Before proceeding with a renal biopsy which of the following blood tests would you order? | Full blood count  International normalised ratio (INR)  Activated partial thromboplastin time (APTT)  Bleeding time  Other |
| 3 | What haemoglobin target should be achieved prior to proceeding with renal biopsy? | >110g/L  >100g/L  >90g/L  >80g/L  >70g/L  no target required  Other |
| 4 | What platelet count should be achieved prior to proceeding with renal biopsy? | >100 x 10⁹ /L  >50 x 10⁹ /L  >20 x 10⁹ /L  no target required  Other |
| 5 | In which circumstance would you give a patient DDAVP prior to renal biopsy? | Never  if eGFR <30  if eGFR <15  Other |
| 6 | How many days before renal biopsy would you withhold the following medications? | |
| 6A | Aspirin in patient with low risk of cardiovascular event | would not withhold  3 days  5 days  7 days  10+ days |
| 6B | aspirin in patient with high risk of cardiovascular event (e.g. recent myocardial infarct, coronary stent) | would not withhold  3 days  5 days  7 days  10+ days |
| 6C | P2Y12 inhibitors (clopidogrel, ticagrelor, etc.) | would not withhold  3 days  5 days  7 days  10+ days |
| 6D | Direct oral anticoagulants (apixaban, rivaroxaban, etc.) | would not withhold  3 days  5 days  7 days  10+ days |
| 7 | When should bridging intravenous heparin be ceased before a renal biopsy? | 4hrs beforehand  6hrs beforehand  10hrs beforehand  12hrs beforehand  Other |
| 8 | When can bridging intravenous heparin be restarted after a renal biopsy? | 6hrs after biopsy  12hrs after biopsy  24hrs after biopsy  48hrs after biopsy  Other |
| 9 | You would prefer renal biopsies to be performed by: | Ultra-sonographers  Senior Renal Registrars  Consultant nephrologists  Radiologists |
| 10 | The maximum number of passes that you would make during a renal biopsy procedure is | 2  3  4  5  6  Other |
| 11 | Which size needle would you most commonly use to perform a renal biopsy, if: | |
| 11A | allograft kidney biopsy | 14 gauge  16 gauge  18 gauge |
| 11B | native kidney biopsy | 14 gauge  16 gauge  18 gauge |
| 12 | Do you order any routine imaging post renal biopsy? | Yes  No |
| 13 | What is an adequate number of glomeruli in a renal biopsy specimen? | 8  12  15  20  Other |
| 14 | In routine renal biopsy, how do you position the patient? | |
| 14A | Native kidney | Supine position  Prone position  Sitting position  Lateral decubitus / lateral recumbent position |
| 14B | Transplant kidney | Supine position  Prone position  Sitting position  Lateral decubitus / lateral recumbent position |
| 15 | After a routine renal biopsy for how long should the patient stay in hospital for observation? | 4 hours  6 hours  12 hours  24 hours  Other |
| 16 | How many renal biopsies would you perform in an average month? | None  1-5  6-10  >10 |
| 17 | What percentage of renal biopsies performed at your centre, would you estimate, have the following complications:   - Decrease in haemoglobin >10g/L - Bleeding requiring transfusion - Bleeding requiring embolisation - Urinary tract infection - Nephrectomy - Death | <1%  1-5%  5-10%  10-25%  25-50%  50-75%  75-100% |
| 18 | When evaluating a patient with an acute kidney injury (AKI), how commonly would you order a renal biopsy in the following situations | |
| 18A | AKI when other investigations are suggestive of acute glomerulonephritis | Rarely  Sometimes  Usually  Always |
| 18B | Presenting as acute glomerulonephritis with elevated ANCA titres | Rarely  Sometimes  Usually  Always |
| 18C | Presenting as acute glomerulonephritis with elevated anti-DNAse titres | Rarely  Sometimes  Usually  Always |
| 19 | In a patient presenting with acute renal failure, after how many weeks of non-recovery would you perform a renal biopsy? | 2 weeks  4 weeks  6 weeks  8 weeks |
| 20 | When evaluating a patient with chronic renal insufficiency (GFR <30 mL/min) of unknown cause, how commonly would you order a renal biopsy in the following situations: | |
| 20A | Patient with normal kidney size on imaging. | Rarely  Sometimes  Usually  Always |
| 20B | Patient with reduced kidney size on imaging. | Rarely  Sometimes  Usually  Always |
| 20C | Patient with haematuria and proteinuria (>1g/day), | Rarely  Sometimes  Usually  Always |
| 20D | Patient with haematuria and <1g/day of proteinuria | Rarely  Sometimes  Usually  Always |
| 21 | When evaluating a patient with normal renal function, how commonly would you order a renal biopsy in the following situations: | |
| 21A | Patient with haematuria and proteinuria (>1g/day) | Rarely  Sometimes  Usually  Always |
| 21B | Patient with proteinuria (>1g/day), without haematuria and normotensive | Rarely  Sometimes  Usually  Always |
| 21C | Patient with proteinuria (>1g/day), without haematuria and hypertensive | Rarely  Sometimes  Usually  Always |
| 21D | Patient with isolated proteinuria >3g/day | Rarely  Sometimes  Usually  Always |
| 21E | Patient with isolated haematuria and normotensive | Rarely  Sometimes  Usually  Always |
| 22 | In a patient with type 2 diabetes mellitus, how commonly would you order a renal biopsy in the following situations: | |
| 22A | Chronic renal insufficiency (eGFR <30 mL/min), without retinopathy | Rarely  Sometimes  Usually  Always |
| 22B | Rapidly deteriorating renal function | Rarely  Sometimes  Usually  Always |
| 22C | Nephrotic range proteinuria | Rarely  Sometimes  Usually  Always |
| 22D | Active urinary sediment | Rarely  Sometimes  Usually  Always |
| 23 | How commonly would you order a renal biopsy on a solitary kidney in the following situations: | |
| 23A | Solitary kidney with abnormal renal function, GFR <30ml/min | Rarely  Sometimes  Usually  Always |
| 23B | Solitary kidney with proteinuria 1-3g/day | Rarely  Sometimes  Usually  Always |
| 23C | Solitary kidney with proteinuria >3g/day | Rarely  Sometimes  Usually  Always |
| 24 | How commonly would you order a renal biopsy in a pregnant mother at <32 weeks gestation, in the following situations: | |
| 24A | GFR <30ml/min of unknown cause | Rarely  Sometimes  Usually  Always |
| 24B | Symptomatic proteinuria >3g/day without clinical features of pre-eclampsia | Rarely  Sometimes  Usually  Always |
| 24C | Symptomatic proteinuria >3g/day with clinical features of pre-eclampsia | Rarely  Sometimes  Usually  Always |
| 25 | How commonly would you order a renal biopsy in a pregnant mother at >32 weeks gestation, in the following situations: | |
| 25A | GFR <30ml/min of unknown cause | Rarely  Sometimes  Usually  Always |
| 25B | Symptomatic proteinuria >3g/day without clinical features of pre-eclampsia | Rarely  Sometimes  Usually  Always |
| 25C | Symptomatic proteinuria >3g/day with clinical features of pre-eclampsia | Rarely  Sometimes  Usually  Always |
| 26 | How commonly would you order a renal biopsy in a transplanted kidney in the following situations: | |
| 26A | Rapid rise in serum creatinine after initially good function, before anti-rejection therapy. | Rarely  Sometimes  Usually  Always |
| 26B | Serum creatinine not improving after anti-rejection therapy. | Rarely  Sometimes  Usually  Always |
| 26C | Slow progressive deterioration in graft function. | Rarely  Sometimes  Usually  Always |
| 26D | New onset proteinuria >3g/day | Rarely  Sometimes  Usually  Always |
